# Comparison of single-nucleotide polymorphisms and microsatellites in inference of population structure

**DOI:** 10.1186/1471-2156-6-S1-S26

**Published:** 2005-12-30

**Authors:** Nianjun Liu, Liang Chen, Shuang Wang, Cheongeun Oh, Hongyu Zhao

**Affiliations:** 1 Department of Epidemiology and Public Health, Yale University, New Haven, CT 06520, USA; 2 Department of Biostatistics, University of Alabama at Birmingham, Birmingham, AL 35294,USA; 3 Department of Molecular, Cellular and Developmental Biology, Yale University, New Haven, CT 06520, USA; 4 Department of Biostatistics, Mailman School of Public Health, Columbia University, New York, NY 10032, USA; 5 Department of Biostatistics, Department of Preventive Medicine, University of Medicine and Dentistry of New Jersey, Newark, NJ 07101, USA; 6 Department of Genetics, Yale University, New Haven, CT 06520, USA

## Abstract

Single-nucleotide polymorphisms (SNPs) are a class of attractive genetic markers for population genetic studies and for identifying genetic variations underlying complex traits. However, the usefulness and efficiency of SNPs in comparison to microsatellites in different scientific contexts, e.g., population structure inference or association analysis, still must be systematically evaluated through large empirical studies. In this article, we use the Collaborative Studies on Genetics of Alcoholism (COGA) data from Genetic Analysis Workshop 14 (GAW14) to compare the performance of microsatellites and SNPs in the whole human genome in the context of population structure inference. A total of 328 microsatellites and 15,840 SNPs are used to infer population structure in 236 unrelated individuals. We find that, on average, the informativeness of random microsatellites is four to twelve times that of random SNPs for various population comparisons, which is consistent with previous studies. Our results also indicate that for the combined set of microsatellites and SNPs, SNPs constitute the majority among the most informative markers and the use of these SNPs leads to better inference of population structure than the use of microsatellites. We also find that the inclusion of less informative markers may add noise and worsen the results.

## Background

Population structure inference from genetic markers is very important in a variety of contexts, such as in admixture and association mapping, evolutionary studies, forensics, medical risk prediction, and wildlife management [1-5]. Statistical methods have been proposed for population structure inference using multilocus genotypes [1,3,5,6] and have been widely used in practice [2,3,5,7].

Single-nucleotide polymorphisms (SNPs) are a class of attractive genetic markers for population genetic studies and for identifying genetic variations underlying complex traits. This is because SNPs are highly abundant, functionally relevant, have relatively low mutation rates, and offer more rapid and highly automated genotyping. With recent efforts to identify SNPs in the human genome, linkage disequilibirum studies in different populations [8,9], and advancements in the efficiency of high-throughput genotyping technology, genome-wide screens of high-density SNPs are becoming increasingly feasible for studies involving a large sample of individuals. However, the usefulness and efficiency of SNPs still need to be demonstrated through large empirical studies, especially in the context of population structure inference, where few studies have been done [4]. To fill in this gap, in this article we compare the information content (informativeness) of SNPs and microsatellites throughout the whole human genome, and compare the performance of SNPs and microsatellites in the context of population structure inference.

## Methods

### Informativeness of markers

There are many measures of marker information content for various purposes [4,10,11]. There is a brief review of measures of marker informativeness for population structure inference in Rosenberg et al. [4]. In this study, we employ the measure of informativeness for assignment (*I*_*n*_) proposed in that article [4].

Consider that there are *i *= 1, 2,..., *K *populations and *m *= 1, 2, ..., *L *loci, with *K *≥ 2 and *L *≥ 1. Locus *m *has alleles *j *= 1, 2, ..., *N*^(*m*)^. The average frequency of allele *j *at locus *m *across the *K *populations is defined as [4]:



where  is the relative frequency for allele *j *of locus *m *in population *i*. The informativeness is defined as [4]:



### Bayesian approach for population structure inference

Pritchard et al. [1,6] introduced a model-based clustering method (STRUCTURE) using multilocus genotype data to infer population structure and assign individuals to populations. They used Bayesian formulation and generated the posterior distribution using a Markov chain Monte Carlo method based on Gibbs sampling. This is the dominant method currently used. We use STRUCTURE 2.0 in our analysis.

### Data

The data we use is the Collaborative Study on the Genetics of Alcoholism (COGA) dataset provided by the Genetic Analysis Workshop 14 (GAW14). There are 328 microsatellites and 16,312 SNPs (4,752 SNPs are provided by Illumina and 11,560 SNPs are provided by Affymetrix) genotyped over all 23 pairs of human chromosomes, including the sex chromosome. A total of 1,614 individuals belonged to eight self-reported ethnic groups: 12 American Indians, 4 Pacific Islanders, 191 Black non-Hispanic, 14 Black Hispanic, 1,074 White non-Hispanic, 78 White Hispanic, 12 others, and 229 without ethnic information. We used all 328 microsatellites and 15,840 "clean" SNPs which are provided by GAW14 with Mendelian errors removed (4,720 SNPs from Illumina and 11,120 SNPs from Affymetrix). From the 1,614 family members, we chose unrelated (founders whose parents are not in the dataset) individuals with both SNPs and microsatellites genotype data. There were 248 individuals left. Among these individuals, there were 4 American Indians, 2 Pacific Islanders, 18 Black non-Hispanic, 3 Black Hispanic, 206 White non-Hispanic, 12 White Hispanic, 2 others, and 1 without ethnic information. In order for each ethnic group to have enough sample size, we finally included 236 unrelated individuals in our study from three ethnic groups: 18 Black non-Hispanic, 206 White non-Hispanic, and 12 White Hispanic.

## Results

A first level of sub-structure (e.g., Black vs. White) was detectable using these data. However, higher orders of sub-structure (e.g., Black non-Hispanic vs. White Hispanic) were not detectable. Nonetheless, we present some results on informativeness comparisons between these higher order sub-structures for illustrative purposes.

### Comparison of information content of microsatellites and SNPs

**Figure 1:**
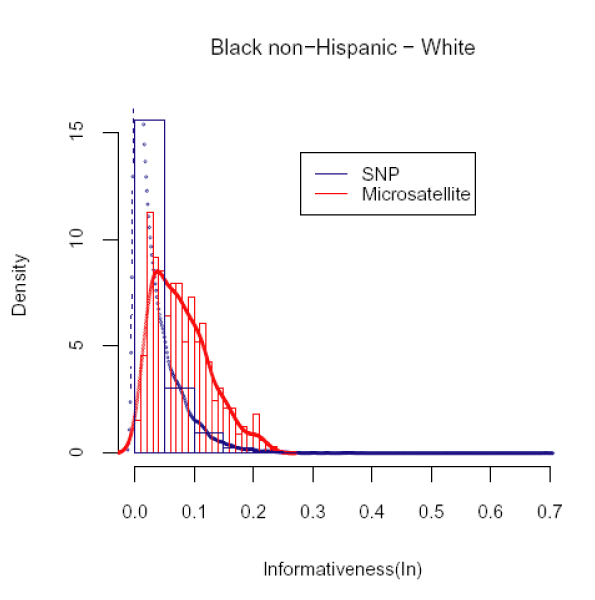
The distributions of informativeness (I_n_) of microsatellites and SNPs for Black non-Hispanic vs. White. The dotted curves are  the estimated kernel densities of the data.

**Figure 2:**
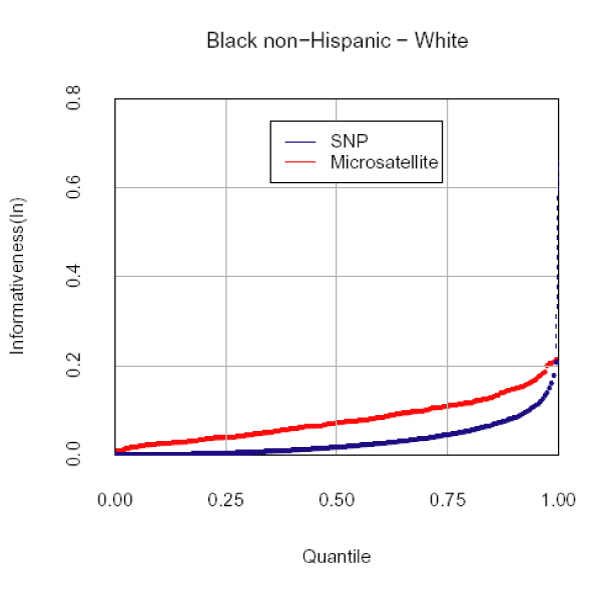
Informativeness quantiles for microsatellites and SNPs for Black non-Hispanic vs. White

Figure 1 shows the distribution of marker informativeness of microsatellites and SNPs for Black non-Hispanic vs. White. The patterns are similar for the distributions between other sets of source populations (i.e., Black non-Hispanic vs. White non-Hispanic, Black non-Hispanic vs. White Hispanic, and White non-Hispanic vs. White Hispanic, figures not shown). On average, microsatellites contain more information than SNPs. This difference is significant (data not shown). For Black non-Hispanic vs. White, random microsatellites have greater informativeness than random SNPs (Figure 2). The same conclusion holds for other sets of source populations (figures not shown). The ratios of the median microsatellite informativeness to median SNP informativeness were 4.17 for Black non-Hispanic vs. White non-Hispanic, 6.84 for Black non-Hispanic vs. White Hispanic, 11.79 for White non-Hispanic vs. White Hispanic, and 4.19 for Black non-Hispanic vs. White (both Hispanic and non-Hispanic). The ratios of the means were 2.47, 3.797, 6.27, and 2.49, respectively, and the 50^th ^percentile of microsatellite informativeness corresponds to the 86^th^, 94^th^, 98^th^, and 86^th ^percentiles of SNP informativeness in the four comparisons. These results are consistent with those observed by Rosenberg et al. [4].

**Figure 3:**
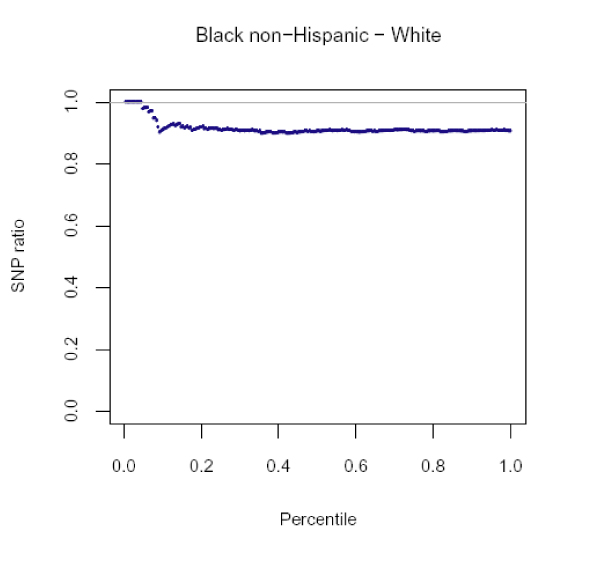
The percentage of SNPs among the most informative markers where microsatellites and SNPs are pooled together (for Black non-Hispanic vs. White).

Figure 3 shows the percentage of SNPs among different numbers of most informative markers (markers with the highest information content values) for Black non-Hispanic vs. White. The patterns were similar for other source populations (figures not shown). Most of the time, SNPs represent the majority among the most informative markers. This is contrary to Rosenberg's observation [4] that "highly informative loci constitute a greater fraction of microsatellites than of SNPs".

### Comparison of performance of microsatellites and snps in inferring population structure

We used STRUCTURE 2.0 [1,6] with all 236 individuals (assuming 2 subpopulations) using the markers with the highest informativeness. For various choices of the number of markers, *M*, five STRUCTURE runs were performed with *M *microsatellites of highest *I*_*n*_(among all microsatellites), and *M *SNPs of highest *I*_*n*_(among all SNPs), respectively. Two individuals with SNP data completely missing were excluded when SNPs were used in the analysis. All STRUCTURE runs employed the admixture model for individual ancestry [1], the *F *model for allele frequency correlations [6], and a burn-in period of length 10,000 followed by 10,000 iterations.

**Figure 4:**
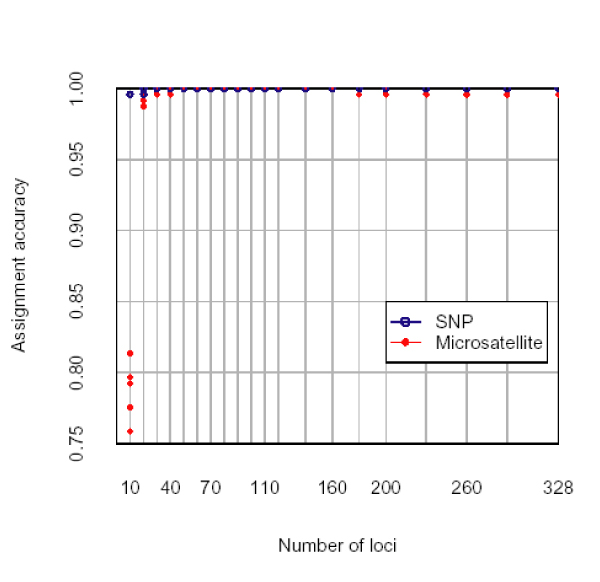
The assignment accuracies for the 5 runs of microsatellites and SNPs each for various numbers of loci.

An individual was considered to be assigned accurately when the greatest proportion of the ancestry identifies the same ethnicity as the pre-defined population group of the individual (by self-identification). Assignment accuracy was defined as the proportion of correctly assigned ethnicities. For each value of *M*, the assignment accuracies of the 5 STRUCTURE runs are shown in Figure 4.

Figure 4 indicates that SNPs of the highest informativeness perform uniformly better than the same number of microsatellites of the highest informativeness, especially when a small number of markers are used. Another finding is that as the number of most informative microsatellites used increases, the result improves. But after a certain value, increasing the number of microsatellites worsens the result. For example, when the top 160 most informative microsatellites are used, all the individuals are assigned correctly to the correct subpopulations. But when all 328 microsatellites are used, one individual was misclassified.

## Discussion

In this article we use COGA data to compare empirically microsatellites and SNPs in the context of population structure inference. Consistent with the findings in Rosenberg et al. [4], we find that, on average, microsatellites are much more informative than SNPs for population structure inference (Figure 1). So a randomly chosen set of microsatellites should have greater informativeness (4 to 12 times) than a random chosen set of SNPs (Figure 2). Our results are based on only two subpopulations, and we expect the difference to be greater when more subpopulations are involved. A surprising finding in our study is that although SNPs are less informative than microsatellites on average, among the most informative markers, SNPs usually constitute the majority (Figure 3). This is inconsistent with the findings in Rosenberg et al. [4]. The main reason may be that there are many more SNPs in our study than in theirs, with 328 microsatellites and 15,840 SNPs, compared with fewer than 400 markers studied by Rosenberg et al. [4]. When we analyze the 4,720 SNPs from Illumina and 11,120 SNPs from Affymetrix separately, we can see that the percentage of SNPs in the most informative markers increases as the total number of SNPs increases (data not shown). Another reason may be that in Rosenberg et al. [4], the individuals and populations in the microsatellite and SNP datasets were different. Thus, we believe that our data may better represent the relative usefulness of SNPs versus microsatellites. Figures 3 and 4 confirm the conclusion of Rosenberg et al. [4] that *I*_*n *_does indeed measure the ability to infer population structure. These two figures indicate that at the right quantity, SNPs can be more informative for population structure inference. Because markers with high informativeness are added first, markers added later have less and less informativeness. Figure 4 indicates that the inclusion of less informative markers may add noise and worsen the results.

The major limitations of our study are that we have only two subpopulations, and the numbers of individuals in the two subpopulations are not balanced. Therefore, studies with more subpopulations and larger and balanced samples are needed to make more thorough empirical comparisons.

Our results used the subjects' self-identification for initial population group classification. Many researchers promote self-identified race/ethnicity as being the most valid measure for most epidemiological studies [12-14]. The National Institutes of Health now requires documentation of minority inclusion on all new grant submissions and considers self-reported race/ethnicity status to be the preferred method of categorization [15]. Gomez et al. [16] showed that accuracy of self-reported ethnicity was high among Blacks and Whites. In our analysis, only Blacks and Whites are used. We conjecture that the self-reported ethnicity should be very accurate.

In conclusion, we have compared microsatellites and SNPs in the context of population structure inference. Although microsatellites are more informative than SNPs in general, our findings show great promise for using SNPs when a large number of SNPs are available.

## Abbreviations

COGA: Collaborative Study on the Genetics of Alcoholism

GAW14: Genetic Analysis Workshop 14

SNP: Single-nucleotide polymorphism

## Authors' contributions

NL participated in the design of the study, performed the analysis, and drafted the manuscript. LC, SW, and CO participated in the design of the study and reviewed the manuscript. HZ conceived the study, participated in its design and helped to draft the manuscript. All authors read and approved the final manuscript.
